# Avirulent *Bacillus anthracis* Strain with Molecular Assay Targets as Surrogate for Irradiation-Inactivated Virulent Spores

**DOI:** 10.3201/eid2404.171646

**Published:** 2018-04

**Authors:** Roger D. Plaut, Andrea B. Staab, Mark A. Munson, Joan S. Gebhardt, Christopher P. Klimko, Avery V. Quirk, Christopher K. Cote, Tony L. Buhr, Rebecca D. Rossmaier, Robert C. Bernhards, Courtney E. Love, Kimberly L. Berk, Teresa G. Abshire, David A. Rozak, Linda C. Beck, Scott Stibitz, Bruce G. Goodwin, Michael A. Smith, Shanmuga Sozhamannan

**Affiliations:** Food and Drug Administration, Silver Spring, Maryland, USA (R.D. Plaut, S. Stibitz); Naval Surface Warfare Center, Dahlgren, Virginia, USA (A.B. Staab, T.L. Buhr, L.C. Beck);; Naval Medical Research Center, Fort Detrick, Maryland, USA (M.A. Munson, J.S. Gebhardt);; US Army Medical Research Institute of Infectious Diseases, Fort Detrick (C.P. Klimko, A.V. Quirk, C.K. Cote, T.G. Abshire, D.A. Rozak);; US Army Edgewood Chemical and Biological Center, Aberdeen Proving Ground, Maryland, USA (R.D. Rossmaier, R.C. Bernhards, C.E. Love, K.L. Berk);; Defense Threat Reduction Agency, Fort Belvoir, Virginia, USA (R.C. Bernhards);; Joint Research and Development, Inc., Stafford, Virginia, USA (C.E. Love, L.C. Beck);; Defense Biological Product Assurance Office, Frederick, Maryland, USA (B.G. Goodwin, M.A. Smith, S. Sozhamannan);; The Tauri Group, Inc., Alexandria, Virginia, USA (S. Sozhamannan)

**Keywords:** Bacillus anthracis, select agent excluded strain, recombinant, genetically inactivated, molecular assay, surrogate, allelic exchange, plasmid, irradiation-inactivated, spore, bacteria, bioterrorism and preparedness

## Abstract

The revelation in May 2015 of the shipment of γ irradiation–inactivated wild-type *Bacillus anthracis* spore preparations containing a small number of live spores raised concern about the safety and security of these materials. The finding also raised doubts about the validity of the protocols and procedures used to prepare them. Such inactivated reference materials were used as positive controls in assays to detect suspected *B. anthracis* in samples because live agent cannot be shipped for use in field settings, in improvement of currently deployed detection methods or development of new methods, or for quality assurance and training activities. Hence, risk-mitigated *B. anthracis* strains are needed to fulfill these requirements. We constructed a genetically inactivated or attenuated strain containing relevant molecular assay targets and tested to compare assay performance using this strain to the historical data obtained using irradiation-inactivated virulent spores.

An effective and constant real-time surveillance capability is crucial for protecting the public from biological threats. Biological threats can be intentional (e.g., resulting from biowarfare or bioterrorism) or unintentional (e.g., resulting from accidental release or emerging infectious diseases) (*1*,*2*). Early detection of a biological threat is critical not only for identifying the threat organism but also for implementing appropriate countermeasures to save and protect the victims and prevent further infection and for decontaminating and reclamating the affected environment and infrastructures.

The bedrock of successful biodetection platforms and sensors is use of well-characterized molecular assays, immunoassays, or other types of detection assays. Any assay development effort requires testing, evaluation, and validation of the assays with live or inactivated spiking materials in appropriate matrices relevant to the environments in which the assays are intended to be used (e.g., aerosol collection filters, soils, or clinical matrices). Distribution and use of select agents and toxins are restricted to facilities that have appropriate approval for storage and use of such materials in containment suites and are regulated by the Federal Select Agent Program of the Centers for Disease Control and Prevention (CDC; Atlanta, GA, USA) and the US Department of Agriculture Animal and Plant Health Inspection Service (Riverdale, MD, USA). For other facilities, inactivated select agents, including inactivated spores, historically were the source of reference materials. Many private and academic organizations, government agencies, and foreign government partners have used these materials for various activities, including quality control exercises and medical countermeasure research.

In May 2015, previously shipped irradiation-inactivated *B. anthracis* spore reference materials were found to contain a small number of live spores (*3*,*4*). The incomplete inactivation of the spores raised concern about the safety and security of these materials and doubts about the validity of the protocols and procedures used to prepare them. After this revelation, the US Department of Defense (DoD) and Department of the Army took a series of measures that included review of existing processes and practices to prepare such reference materials (*5*); placement of a moratorium on shipping of inactivated *B. anthracis* and other select agents from DoD laboratories until further review (*6*); formation of an independent entity (BSAT Biorisk Program Office) to oversee all Biologic Select Agents and Toxins (BSAT) activities within DoD; and implementation of various recommendations of different committees established to evaluate BSAT risk mitigation strategies (*5*,*7*,*8*).

Currently, guidance for implementing the Secretary of the Army directive 2016-24 for the DoD BSAT biosafety program (*7*) has been drafted, with many new measures put in place for the safe handling of BSAT and BSAT-derived products within DoD laboratories and transfer and tracking of such materials across agencies and laboratories. One of the 3 key activities identified in this directive is to explore safer alternatives to BSAT, inactivated BSAT, and BSAT derivatives to reduce health and safety risks associated with BSAT production, handling, and distribution (*7*).

We describe the construction and characterization of a safer alternative to regulated *B. anthracis*: a genetically inactivated (rather than irradiation-inactivated) avirulent *B. anthracis* strain into which specific nucleic acid assay targets for pXO1 and pXO2 replicons have been introduced. The resulting recombinant strain substitutes for and reacts similarly to regulated *B. anthracis* in molecular testing, whereas currently excluded strains (such as Sterne) lack the pXO2 target (Technical Appendix Figure 1). The resulting recombinant strain can be used for testing PCRs used in many biodefense programs. Also, we demonstrate that these spores can be further inactivated by irradiation so they can be used even in a Biosafety Level (BSL) 1 setting.

**Figure 1:**
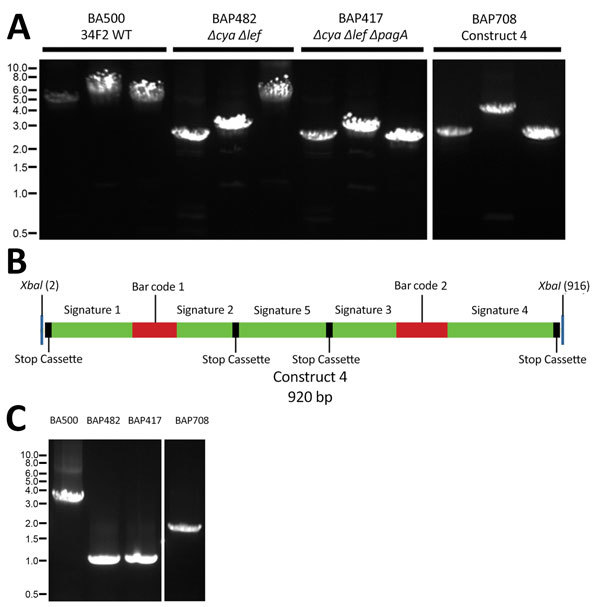
Verification of toxin gene deletions and the genetic structure of the construct 4 cassette in *Bacillus anthracis* surrogate strain. A) PCR verification of toxin gene deletions in BA500 (Sterne 34F2) derivatives. Single colonies were processed and used as templates for PCR with respective primers as described in Methods. For each strain, primers were used to amplify, from left to right, the regions of *cya* (SS2166/SS2167), *lef* (SS2164/SS2165), and *pagA* (SS2168/SS2169) on pXO1. B) Schematic representation of BAP708 (construct 4) cassette. Green bars represent the PCR signatures, red bars represent bar codes, and black boxes represent stop codons in all 3 open reading frames. *XbaI* sites at the ends of the cassette used in subcloning of the insert are marked. C) PCR verification of the presence of construct 4 synthetic sequence cassette in BAP708, using primers immediately flanking the *lef* deletion region (RP214 and RP215). Strains and PCR primers are listed in Table 1. Ladder indicates size in kbps. WT, wild-type.

## Materials and Methods

### Strains, Plasmids, and Primers

*Escherichia coli* and *B. anthracis* strains used in this study are listed in Table 1. The plasmids used in various cloning steps and the primers used for amplification, sequence verification, and diagnosis of constructs also are listed in Table 1.

**Table 1:** Genotypic characteristics of *Escherichia coli* and *Bacillus anthracis* strains and plasmids and primers used to determine avirulent *B. anthracis* strain with molecular assay targets

Strain, plasmid, primer	Genotype	Reference/source
Strain		
DH5α	*F^−^ Φ80lacZΔM15 Δ(lacZYA-argF) U169 recA1 endA1 hsdR17(rk^−^, mk^+^) phoA supE44 thi-1 gyrA96 relA1 λ^−^*	Laboratory collection
SCS110	*rpsL thr leu endA thi-1 lacY galK galT ara tonA tsx dam dcm supE44 Δ(lac-proAB)*	Stratagene (La Jolla, CA, USA)
SM10	*thi thr leu tonA lacY supE recA::RP4–2-Tc::Mu KmR λpir*	(*9*)
S17.1	*hsdR pro recA, RP4–2 in chromosome, Km::Tn7 (Tc::Mu)*	(*9*)
DH5α/pSS1827	*F^−^ Φ80lacZΔM15 Δ(lacZYA-argF) U169 recA1 endA1 hsdR17(rk^−^, mk^+^) phoA supE44 thi-1 gyrA96 relA1 λ^−^, pSS1827 (Replicon fusion of pBR322 and pRK2013 at EcoRI and SalI sites)*	(*10*)
BA500	*B. anthracis* Sterne 34F2	Laboratory collection
BAP482	BA500 *Δcya* and *Δlef* (double toxin deletion)	(*11*)
BAP417	BA500 *Δcya, Δlef, and ΔpagA* (triple toxin deletion)	(*11*)
BAP708	BA417 with construct 4 (Signatures 1–5)	This study
Plasmid	Description	Source
pT7 Blue	Cloning vector	Novagen-MilliporeSigma, St. Louis, MO, USA
pT7 Blue::4	Construct 4 (Signatures 1–5)	This study
pRP1091	*Δlef* derivative of shuttle vector pRP1028	(*11*)
Primer	Sequence, 5′ → 3′	Application
RP411	TTTCACACAGGAAACAGCTATGACC	Amplify constructs
RP645	CCAGTCACGACGTTGTAAAACGAC	Amplify constructs
SS2178	GTAAATTATTTAGCAAGTAAATTTTGGTG	Sequence constructs
RP214	TATGGTCTCGGATCCTTTGGCTTTAACGAAATGTATGTGC	Diagnose, sequence *lef*
RP215	TATGGTCTCCGGCCGTTTCAGTTATTCATTCTGGATAGTC	Diagnose, sequence *lef*
SS2164	CACGAGAAGAGTATTTAAAGAAAATC	Diagnose *lef*
SS2165	AACTATAGGACAATATTCATTACCATG	Diagnose *lef*
SS2166	ATATCAAGTTTAATTGTTAAGTTTGAAGG	Diagnose *cya*
SS2167	CCCGCGGCCGCAACCAAATGGTTTTCATTTCTTAG	Diagnose *cya*
SS2168	CGCATATAAGCAAATACTTAATTGGTC	Diagnose *pagA*
SS2169	GGATAGGGTTTAACAACTTAATAATCCC	Diagnose *pagA*

### Synthesis of a Recombinant Plasmid Carrying PCR Signatures

We synthesized the recombinant construct 4 cassette, containing 5 different PCR signatures, commercially (Blue Heron, LLC, Bothel, WA, USA) and cloned into pT7Blue (Novagen-MilliporeSigma, St. Louis, MO, USA). The cassette was sequence-verified and PCR-amplified from this plasmid. The PCR product and a *lef* deletion plasmid pRP1091 (*11*) were digested with *Xba*I, ligated, and transformed into TOP10 *E. coli* cells (Invitrogen). Successful cloning of the insert was confirmed by restriction enzyme digestion, PCR, and sequencing.

### Construction of Tagged *B. anthracis* Sterne Triple Knockout Strain

We conducted transfer and integration of the cloned insert by allelic exchange as described previously (*11*) (Technical Appendix Figure 2). We designated the final construct recombinant *B. anthracis* Surrogate with Assay Targets (rBaSwAT-BAP708), hereafter referred to as BAP708.

**Figure 2:**
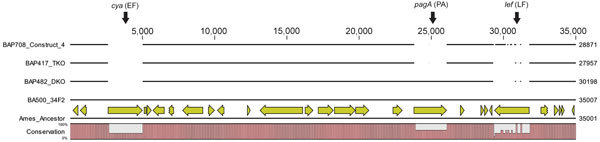
Whole-genome sequence verification of the deletion of toxin genes in *Bacillus anthracis* Sterne 34F2 derivatives. Comparative genomic view of the ≈35-kbp region of the pXO1 containing the toxin genes *cya*, *pagA,* and *lef* is shown. The bottom line indicates the sequence of Ames ancestor along with the annotations. Conservation of the same genetic structure in the grandparent strain BA500 is indicated. Deletions in the parent strains (DKO and TKO) and construct 4 are indicated by breaks in the lines and in the conservation percentage index at the bottom. DKO, double knockout; TKO, triple knockout.

### PCRs

#### Verification of Toxin Gene Deletions and Presence of Synthetic Cassette

We resuspended single colonies of the strains in 50 μL of PCR-Lyse (Epicentre) or Y-PER (Thermo Fisher Scientific, Waltham, MA, USA), vortexed, and incubated them at 99°C for 15 min. Five μL of each lysate was used as a template for PCR (50 cycles), with 2.5 μL of each 10 μM primer and 0.5 μL of Phusion polymerase (Thermo Fisher Scientific) in 50-μL reactions. Annealing temperatures were 49°C (primers RP214/RP215 [*lef*] and SS2166/SS2167 [*cya*]), 54°C (primers SS2168/SS2169 [*pagA*]), and 59°C (primers SS2164/SS2165 [*lef*]) (Table 1). Five μL of each PCR product was run on a 0.8% ethidium bromide agarose gel.

#### Verification of PCR Signature Sequences

We streaked *B. anthracis* strains on tryptic soy agar plates for isolation and incubated them overnight at 37°C before inoculating a colony from each strain into 15 mL of 3% brain heart infusion and incubating cultures with shaking (100 rpm) for 24 h at 37°C. We then centrifuged the entire culture to pellet the cells (room temperature, 10 min, 2,000 × *g*) and extracted DNA using the MoBio Ultraclean Microbial DNA Isolation Kit (QIAGEN Inc., Germantown, MD, USA) according to the manufacturer’s recommended protocol; we eluted DNA in a volume of 200 μL. DNA concentration was determined using a NanoDrop (Thermo Fisher Scientific). We diluted extracts such that PCR reactions were performed starting with either 10 or 50 genomic copies. Various *B. anthracis*–specific PCRs were conducted on an ABI 7500 or 7900 instrument (*12*).

### Animal Study to Evaluate Pathogenicity of the Recombinant Strain

We made spore preparations of various strains using published protocol (*13*,*14*). We infected female A/J mice (6–8 weeks old; Charles River, Frederick, MD, USA) subcutaneously with Sterne (34F2) and Sterne derivative spores and checked the mice daily for clinical signs. Animal research at the United States Army Medical Research Institute of Infectious Diseases was conducted under an animal use protocol approved by the Institute’s Institutional Animal Care and Use Committee in compliance with the Animal Welfare Act, Public Health Service Policy, and other federal statutes and regulations relating to animals and experiments involving animals. The facility where this research was conducted is accredited by the Association for Assessment and Accreditation of Laboratory Animal Care International and adheres to principles stated in the Guide for the Care and Use of Laboratory Animals (https://grants.nih.gov/grants/olaw/Guide-for-the-Care-and-use-of-laboratory-animals.pdf).

### Large-Scale Spore Preparation

We produced BAP708 spores according to the protocol described (Technical Appendix) (*15*–*19*), and determined spore counts after heat inactivation to kill any viable vegetative bacteria. We assessed the quality of the spores (particle size and uniformity, diameter, and particle number) using a Coulter counter. In addition, we conducted phase contrast microscopy to examine the uniformity in size of spores and absence of spore clumps. Sporulation efficiency is the ratio of total CFUs before heat inactivation to CFUs after treating the culture at 65°C for 30 min.

### Irradiation Inactivation of Spores and Postirradiation Sterility Testing

We irradiated 60 mL of the spores in a JL Shepperd-Model 109–68 Cobalt 60 instrument at a rate of 10,975 rads/min for a total of 456 min, with a final dose of ≈5 × 10^6^ rads (50 KGreys). We tested complete inactivation and loss of viability of the spores using the recently established CDC-recommended protocol for select agent spores (*20*). We inoculated 6 mL (10%) of the inactivated spore preparation into 60 mL of Terrific broth and incubated at 37°C for 7 d, and plated 1.2 mL (200 μL × 6 plates; i.e., 2% of the culture volume) on Mueller-Hinton agar and incubated for an additional 7 d. No growth was found on any of the plates. We used positive (unirradiated BAP708) and negative (uninoculated broth of the same type and volume under test) controls to ensure the validity of the protocol.

### Phage Sensitivity

We tested for phage sensitivity as described using the spot titer method (*21*). In brief, a bacterial lawn of test strains was prepared using log phase cultures and 10 μL of various dilutions of phages AP50 and γ were spotted on the lawn and incubated overnight at 37°C. 

### Comparison of Assay Performance of BAP708 Spores to Historical Data from Irradiation-Inactivated Select Agent *B. anthracis* Spores

We prepared liquid and filter extracted samples according to established protocols using 2 separate aliquots of live and irradiation-inactivated BAP708 spore preparations. We diluted spore stock (≈2.0 × 10^10^ CFU/mL) in 1× phosphate-buffered saline to a spiking concentration of 2.0 × 10^6^ CFU/mL and either extracted the stock directly as liquid samples or spiked it onto quarter filters, allowed to it dry, and extracted it as filter samples. We used both clean and simulated dirty filters. We extracted samples in accordance with an established single-tube extraction protocol using Amicon Ultra −0.5 Centrifugal filter devices (MilliporeSigma). In brief, we extracted DNA by mechanical disruption using a bead beater (*22*) and size exclusion filtration and eluted results in a volume of 200 μL. We heat treated DNA extracts to inactivate any nuclease (65°C, 10 min) before use in PCR analysis. We used 5 μL of DNA in 5 different *B. anthracis*–specific real-time PCRs on the ABI 7500 or ABI 7900 platform (*12*).

### Lateral Flow Immunoassay

We tested live and inactivated spores in a standard lateral flow immunoassay (LFI) that is designed to detect *B. anthracis* spores (S. Sozhamannan, unpub. data). We used 100 μL of spores in each test and quantified the intensities of test and control lines using a thin layer chromatography scanner and software for scanning LFIs (CAMAG-TLC-3). We plotted results as relative absorbance units versus concentration of spores, set the background threshold at 30 scanner units, and scored all results >30 units as positive. The measurements were done in quadruplicate, and the minimal spore concentration that crossed the threshold was reported as the limit of detection using each spore preparation.

## Results

### Rationale for Construction of Recombinant Strains with Assay Targets

Mitigating the risk associated with irradiation-inactivated wild-type *B. anthracis* strains, such as Ames, required use of avirulent, excluded strains as reference materials for detection/diagnostic assay developmental efforts. However, assay targets for virulent strains most often are located in genes that are absent in the excluded strains. *B. anthracis* detection relies on 3 specific markers, 1 each on the chromosome and the pXO1 and pXO2 replicons. Strains containing plasmid pXO2 are classified as select agents (*23*), and Sterne lacking pXO2 but carrying pXO1 can be pathogenic for some mice strains because of the presence of the toxin genes (*pagA*, *lef*, and *cya*) on pXO1 (*24*). Strains lacking either pXO1 or pXO2 lack target(s) for the missing plasmid and hence are of limited utility as reference materials. Therefore, we decided to construct recombinant strains carrying all 3 assay targets in the background of a highly attenuated excluded strain. We chose a Sterne derivative, designated ΔSterne triple knockout strain (BAP417), in which all 3 toxin genes have been deleted (Technical Appendix Table) (*11*) and that lacks both pXO1 and pXO2 assay targets (Figure 1, panel A), as confirmed by whole-genome sequence analyses (Figure 2). In this strain, assay signatures for pXO1 and pXO2 plasmids were introduced into the ΔpXO1 backbone as described in Materials and Methods.

### Synthesis of Assay Target Cassette and Transfer of the Cassette into *B. anthracis*

Of 4 constructs made, in synthetic construct 4 described here, 5 signatures (PCR targets; i.e., amplicon sequences, including primer and probe sequences) and 2 bar codes were embedded. The bar codes are unique for each construct and can be used to track the strain and distinguish it from the wild type. In addition, stop codons in all 3 open reading frames were placed on the 5′ and 3′ ends of the cassette to prevent any fortuitous translation of the inserts from read-through from neighboring transcriptional signals (Figure 1, panel C).

We conducted transfer of the cassettes onto *B. anthracis* ΔpXO1 as described previously (*11*). We determined the characteristics and predicted phenotypic properties of the resulting final scarless construct (Table 2). The deletion-insertion was verified by PCR (Figure 1, panels A,C) and further confirmed by whole-genome sequence analyses (Figure 2).

**Table 2:** Description of the DNA inserts in the recombinant *Bacillus anthracis* surrogate strain*

Replicon of assay target	WT gene	Size, bp	Assay	WT insert size, bp (%)†	Mutant insert size, bp‡	Predicted biological characteristic of recombinant product	Antimicrobial resistance/sensitivity of recombinant product
pXO2	*capA*	1,236	Signature 1	98 (7.9)	98	NC (Tox^−^Cap^−^)	Spc^S^, Kan^S^, Amp^S^
pXO1	*pagA*	2,295	Signature 2	110 (4.8)	113	NC (Tox^−^Cap^−^)	Spc^S^, Kan^S^, Amp^S^
pXO1	*pagA*	2,295	Signature 3	137 (6.0)	142	NC (Tox^−^Cap^−^)	Spc^S^, Kan^S^, Amp^S^
pXO2	*capB*	1,395	Signature 4	182 (13.0)	186	NC (Tox^−^Cap^−^)	Spc^S^, Kan^S^, Amp^S^
pXO1	*pagA*	2,295	Signature 5	153 (6.67)	NA	NC (Tox^−^Cap^−^)	Spc^S^, Kan^S^, Amp^S^
Bar code 1	NS	78	NA	NA	NA	NA	NA
Bar code 2	NS	90	NA	NA	NA	NA	NA

*Amp, ampicillin; Cap^–^, capsule negative; Kan, kanamycin; NA, not applicable; NC, no change from parent; NS, nonbiological, nonprotein-coding sequence; S, sensitive; Tox^–^, protective antigen (PA), lethal factor (LF), and edema factor (EF) negative; Spc, spectinomycin; WT, wild-type. †Percentage of the wild-type gene. ‡Contain unique restriction sites.

### Characterization of the Recombinant Strain

We conducted a comprehensive phenotypic and genotypic characterization of the recombinant strain, BAP708, to establish its avirulent phenotype and the presence of assay targets for both molecular and immunoassays (Table 3). The characterization included basic microbiological tests, such as colony morphology on selective agar plates; biochemical and phage sensitivity tests; molecular assays, such as PCR; immunoassays, such as LFI; whole-genome sequencing; and animal lethality.

**Table 3:** Characterization of *Bacillus anthracis* surrogate strains*

Strain	Inserts	Summary of assays performed								
WGS accession nos.	Toxin deletion PCR	Cassette PCR	Signature PCR†	Spore LFI	Phage test AP50	Phage test γ	Sporulation percentage	Animal model lethality
BA500	None	NRIZ00000000	WT	NI	ER	+	Sensitive	Sensitive	Normal (≈100%)	Lethal
BAP482	None	NRJA00000000	ED	NI	ER	+	Sensitive	Sensitive	Normal (≈100%)	Nonlethal
BAP417	None	NRJB00000000	ED	NI	ER	+	Sensitive	Sensitive	Normal (≈100%)	Nonlethal
BAP708	Signatures 1–5	Yes	ED	EI	ER	+	Sensitive	Sensitive	Normal (≈100%)	Nonlethal
Ames	None	CP009979–CP009981	WT	WT	ER	+	Sensitive	Sensitive	Normal (*25*) (≈100%)	Lethal (*26*)
*B. thuringiensis* Al Hakam	None	CP009645–CP009651	NP	NI	ER	−	Resistant	Resistant	Normal (*16*) ≈100%)	ND

*ED, expected deletion; EI, expected insert; ER, expected result; LFI, lateral flow immunoassay; ND, not done; NI, no insert; NP, no PCR product; WGS, whole-genome sequence; WT, wild-type; +, positive; –, negative. †Results in Table 4.

### PCR Analyses of Toxin Gene Deletions and Presence of the Cloned Cassettes

We conducted PCRs to confirm the toxin gene deletions and the presence of the cassette in BAP708. We used primers flanking the toxin genes as well as the insertion site (*11*) to amplify the region. The double (*pagA*) and triple knockout strains showed the expected deletions, and BAP708 showed an increase in fragment size corresponding to cassette insertion at the expected location (*lef*) (Figure 1, panels A, B). PCR products of expected sizes were obtained using DNA from the regulated strain *B. anthracis* Ames, whereas no products were obtained using DNA from *B. thuringiensis* Al Hakam, indicating absence of the toxin genes. In addition, a real-time PCR designed to distinguish this strain from wild-type virulent strains, such as Ames, detected BAP708 exclusively (data not shown).

### Whole-Genome Sequencing and Analysis

We used Illumina next-generation sequencing technology to produce whole-genome sequences of various strains. Whole-genome sequences of the 3 parental strains have been deposited in GenBank under accession nos. BA500-NRIZ00000000, BA482-NRJA00000000, and BA417-NRJB00000000 (*27*). Analysis of the ΔpXO1 toxin region indicated that the triple knockout strain (BAP417) and its derivative (BAP708) lacked the *pagA*, *lef*, and *cya* genes that encode the 3 anthrax toxin subunits (Figure 2).

### Sporulation

The infective form of *B. anthracis* is the spore, not the vegetative cell. Many detection/diagnostic assays target spore antigens (*28*). For immunoassays, the antigenic epitopes are most likely spore coat proteins, although there are immunoassays against anthrax toxin, which is produced by vegetative cells and secreted into the extracellular milieu (*29*). For nucleic acid–based tests, DNA extracted from spores is used as template to detect *B. anthracis*. Therefore, we assessed the spore-forming ability of BAP708. 34F2 and its derivative BAP708 produced spores efficiently (efficiencies ≈100% [Table 3]). The final titer for the BAP708 spore preparation was ≈1.5 × 10^10^ spores/mL, and the particle size was 1.153 ± 0.122 μm. Sporulation results for regulated *B. anthracis* Ames strain and the negative control *B. thuringiensis* Al Hakam strain have been published and were normal (*16*,*25*).

### Phage Sensitivity

One diagnostic test recommended by CDC for the suspected presence of *B. anthracis* in a sample is sensitivity of the bacterial isolate from the sample to γ phage. AP50c is another phage that can be used to verify *B. anthracis* (*30*). The recombinant strain, BAP708, exhibited sensitivity to both phages, as did the parent strains (Table 3), although they were less sensitive than other strains, such as Sterne 7702 (data not shown). In addition, bacteria in log phase were much more sensitive to infection by AP50c than were stationary phase cells (data not shown), which may be due to phase-dependent expression of the AP50c phage receptor, Sap (*31*,*32*). Regulated *B. anthracis* Ames strain was sensitive to both phages, and the negative control *B. thuringiensis* Al Hakam strain was resistant to both phages.

### Molecular Assays

We assessed the performance of BAP708 in molecular assays (Table 4). Real-time PCRs using BAP708 and *B. anthracis* Ames produced expected results in accordance with the assay targets present or introduced into the strain, whereas assays using the negative control *B. thuringiensis* Al Hakam strain did not produce a positive amplification.

**Table 4:** Real-time PCR signature analyses in various *Bacillus anthracis* strains

Strain	Material type	Insert	Molecular assay result					
Chr	Sig 1	Sig 2	Sig 3	Sig 4	Sig 5
BA500	Vegetative cells†	None	+	−	+	+	−	+
BAP482	Vegetative cells†	None	+	−	+	+	−	+
BAP417	Vegetative cells†	None	+	−	−	−	−	−
BAP708	Vegetative cells†	Signatures 1–5	+	+	+	+	+	+
Ames‡	Vegetative cells†	Wild type	+	+	+	+	+	ND
*B. thuringiensis* Al Hakam	Vegetative cells†	None	−	−	−	−	−	ND
BAP708	Live spores-liquid extract	Signatures 1–5	+	+	+	+	+	ND
BAP708	Live spores-filter extract	Signatures 1–5	+	+	+	+	+	ND
BAP708	Inactivated spores-liquid extract	Signatures 1–5	+	+	+	+	+	ND
BAP708	Inactivated spores-filter extract	Signatures 1–5	+	+	+	+	+	ND
Ames‡	Inactivated spores-liquid extract	Wild type	+	+	+	+	+	ND
Ames‡	Inactivated spores-filter extract	Wild type	+	+	+	+	+	ND
*B. thuringiensis* Al Hakam	Live spores-liquid extract	None	−	−	−	−	−	ND
*B. thuringiensis* Al Hakam	Live spores-filter extract	None	−	−	−	−	−	ND

*Chr, chromosomal marker; ND, not done; Sig, signature; +, cycle threshold <35; –, cycle threshold >35. †Genomic DNA extracted from vegetative cells was used as template for PCR (equivalent to 10 and 50 copies or 100 and 500 copies for *B. thuringiensis* Al Hakam). ‡Historical data.

### Validation of Avirulent Nature of Recombinant Strain

**Figure 3:**
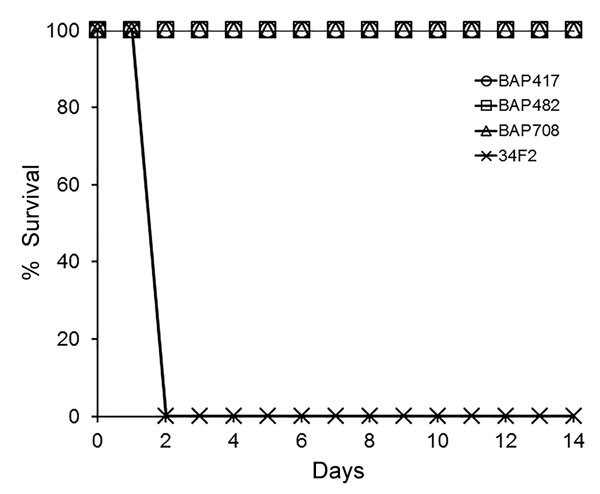
Role of *Bacillus anthracis* toxin components in lethality of Sterne strain 34F2 in female A/J mice and nonlethality of *B. anthracis* Sterne derivatives. Groups of mice were infected subcutaneously with *B. anthracis* spores of Sterne strain BA500 (34F2) or isogenic strains deficient for different toxin genes TKO-BAP417 (*Δcya, Δlef, ΔpagA*); DKO-BAP482 (*Δcya, Δlef*); BAP708- construct 4 (*Δcya, Δlef, ΔpagA*) plus insert. Fifty percent lethal dose equivalents ranged from ≈106 to 165. Based on a 1-sided Fisher exact test, p = 0.0003 for all groups (N = 10) versus the control 34F2 (N = 5) group.

We inoculated female A/J mice (6–8 weeks old) subcutaneously with spores of Sterne (34F2) or its derivative. The 50% lethal dose (LD_50_) in this model is 1.1 × 10^3^ *B. anthracis* Sterne (pXO1^+^/pXO2^−^) spores (*33*), and the LD_50_ of fully virulent strains, such as Ames, and other species of *Bacillus,* such as *B. cereus* G9241, have been reported (*26**,**33**,**34*). The calculated delivered LD_50_ equivalents are as follows: BAP417, 109.7; BAP482, 152.7; BAP708, 106.1; and 34F2, 164.8. The animals were monitored daily for clinical signs for up to 14 days. Only the mice challenged with 34F2 showed any signs of disease; these mice succumbed to the infection or were euthanized after meeting early-endpoint criteria within 48 h (Figure 3). All animals in the other groups showed no signs of disease, indicating the avirulent nature of the toxin gene deletion derivatives.

### Comparisons of Assay Performance of Recombinant Strain to Wild-Type and Inactivated Wild-Type Spores

We tested live and inactivated BAP708 spores for performance in immunoassays and PCRs to evaluate the effect of irradiation on assay targets. Unlike the near neighbor *B. thuringiensis* Al Hakam, BAP708 spores reacted positively in LFI, albeit weakly compared with historical reference materials, such as inactivated *B. anthracis* Ames (data not shown). PCR was done on DNA extracted in 2 different formats: liquid and spiked filter. All extracts reacted as expected in PCRs. These results were comparable to historical data obtained using irradiation-inactivated Ames spores. The inactivated *B. thuringiensis* Al Hakam spores treated and extracted similarly did not yield any positive results (Table 4).

## Discussion

There are multiple instances of poor biosafety/biosecurity measures or laboratory accidents resulting in the release of harmful pathogens (*4*,*35*–*37*). These incidents underscore the lack of knowledge about factors influencing environmental survival of biological agents, the steps needed to ensure that established biosafety methods continue to work and meet expectations, and the need to acquire knowledge about how to recognize early any failure in established laboratory methods over time (*1*). We demonstrated an alternate approach that can potentially minimize risks associated with using BSATs and perhaps eliminate their use in some applications.

The need for BSATs and their derivatives for research and countermeasure development is inevitable. Guaranteeing inactivation of BSATs, especially spores, without adversely affecting their diagnostic and therapeutic targets can be problematic. However, the strategy described here of genetically inactivating the organism to mitigate the risk is a safer approach. In this study, we chose a *B. anthracis* strain that carries one of the virulence plasmids (pXO1) and removed the toxin genes from that plasmid to make it completely avirulent. Another option would have been to introduce pXO1 and pXO2 assay targets into the chromosome in a pXO1^−^ and pXO2^−^ background. However, the copy numbers of pXO1 and pXO2 have been determined to be slightly higher than that of the chromosome (1, 2, and 4 copies for the chromosome, pXO2, and pXO1 respectively) (*38*). To maintain a slightly higher copy number of the introduced plasmid assay targets, we introduced the assay targets into the ΔpXO1 backbone rather than into the chromosome in a strain lacking both pXO1 and pXO2. This way, assay results would be comparable in terms of copy numbers and cycle threshold values to historical assay data produced from a strain such as Ames.

In introducing the assay targets, neither full-length genes nor any antibacterial drug marker were introduced. Moreover, the surrogate strain is similar to virulent *B. anthracis* with respect to its utility as a reference material, except that it is risk-mitigated. In addition, unique bar codes have been introduced to distinguish the surrogate from the wild-type virulent agent and for forensic purposes.

The approach we describe can be easily adapted for other assay targets and applications. For example, genes encoding vaccine antigens, such as nonlethal variants of toxin genes, could be cloned and expressed in the recombinant strain. Because it is a platform technology, it would be relatively easy to construct strains for other assays by exchanging assay targets, which would also be safer and more cost-effective than handling BSATs and their derivatives. Noninfectious virus-like particles carrying assay targets could be created for BSL3 and BSL4 viral agents (*39*,*40*). The major disadvantage to this approach is that for every new assay signature/target, a new strain needs to be constructed, which may entail initial investment of time and funds to create the framework. Another disadvantage is that not all applications can be fulfilled by any 1 strain.

BSATs and inactivated BSATs pose risk and cost with respect to safety and security in production, validation, and shipping. Genetically inactivated and modified organisms provide almost the same level of assay capabilities as BSAT agents but with greatly reduced risk and cost. In addition, the recombinant construct described here is excluded from any regulatory concerns, such as need for exclusion from CDC select agent experiments, recombinant DNA advisory committee guidelines, or International Biological Weapons Convention regulations. Therefore, development of risk-mitigated solutions, such as the one we describe, can help minimize and perhaps prevent mishaps, such as the incident that came to light in 2015.

Technical Appendix Additional methods and results for development of avirulent *Bacillus anthracis* strain and schematic representations of wild type, various mutants, the recombinant surrogate strain, and allelic exchange.
