# Supplementary material for: Avirulent Bacillus anthracis Strain with Molecular Assay Targets as Surrogate for Irradiation-Inactivated Virulent Spores
Source: Emerg Infect Dis. 2018 Apr;24(4):691–9. doi: 10.3201/eid2404.171646 (PMC5875273; doi:10.3201/eid2404.171646)
Supplement: Technical Appendix — Additional methods and results for development of avirulent Bacillus anthracis strain and schematic representations of wild type, various mutants, the recombinant surrogate strain, and allelic exchange. [file 17-1646-Techapp-s1.pdf]

Article DOI: http://dx.doi.org/10.3201/eid2404.171646

Avirulent *Bacillus anthracis* Strain with Molecular Assay Targets as Surrogate for Irradiation-Inactivated Virulent Spores

Technical Appendix

Large-Scale Spore Preparation from *B. anthracis* BAP708 Strain

Methods

Bacillus anthracis BAP708 was used to make a large-scale preparation of spores. Sporulation medium was 2.5% nutrient broth amended with CCY salts (*1*–*4*) at pH 7.0. CCY salts were originally described as a mineral mixture for amending of a nonanimal-derived medium consisting of casein-acid hydrolysate, casein-enzyme hydrolysate, and yeast extract medium (*5*). Nutrient broth 2.63% and 30× KPO_4 (potassium phosphate) buffer (CCY buffer) were autoclaved as independent components. CCY divalent cations were sterile-filtered and stored at –80°C. Nutrient broth and CCY buffer were combined before addition of CCY divalent cations to mitigate divalent cation-phosphate precipitation. Tryptic soy agar was streaked with frozen samples provided by the Defense Biological Product Assurance Office (Frederick, MD, USA). After incubation for 16 ± 2 h at 37°C, a single colony from a tryptic soy agar plate was transferred to 10 mL of sporulation medium (preheated to 37°C) and vortexed for 30 s. Pre-aerated and preheated sporulation medium (200 mL medium in 1–1 baffled Corning Erlenmeyer flasks with filter caps) was inoculated with 0.6 mL from the 10 mL of inoculum. The Erlenmeyer flasks were then incubated at 34°C with shaking (300 rev min^{-1}) for 72 ± 2 h in a New Brunswick Scientific shaker/incubator (Eppendorf, Hauppauge, NY, USA). Sporulated cultures were amended with 35.5 mL of 20% Tween 80 (final concentration 3%) and incubated an additional 24 ± 2 h, 34°C at 300 rev min^{-1} to disperse (“unclump”) spores. Spores were harvested by centrifugation at 2,000 × g, 20°C for 10 min. Spores were washed twice with 200 mL of 3% Tween 80 at room temperature (22 ± 4°C) for 24 ± 2 h at 200 rev min^{-1}. Spores were resuspended in 10–20 mL of 0.1% Tween 80 and then characterized by heat-resistant titers, light

microscopy and Coulter analysis (*3,6,7*). Coulter analysis was used to assess spore clumping, determine spore size, and quantify spore cleanliness. Blood agar plating showed no β hemolysis.

Results

With the specified sporulation methods, *B. anthracis* BAP708 strain surpassed the quality/quantity criteria (“sporulation thresholds or requirements”). BAP708 spore preparations surpassed the threshold titer of 1e8 spores/ml of sporulation medium (objective was 1e9 spores/mL of sporulation medium) before spore harvest and purification. The spore preparations were not heat shocked, but aliquots were removed for heat shocking. An aliquot of at least 1e7 spores from each spore preparation showed heat resistance (65°C, 30 min) using a standard quantitative tryptic soy agar plate assay. The BAP708 spore mode size was 1.0–1.5 µm volume-equivalent spherical diameter after measuring at least 500 spores using a Beckman Coulter Multisizer (Beckman Coulter, Indianapolis, IN, USA). Macrobacillus spores tend to agglomerate and stick to surfaces (*8*) due to spore hydrophobicity (*9–13*) and can be specifically attributed to the exosporium that is absent in microbacillus species, such as *B. atrophaeus*, commonly known as Bg (*4*,*14*). Spore suspension and spore dilution in 0.1% of the nonionic surfactant Tween 80 was used to reduce spore sticking and improve lab-to-lab quantitation as previously published (*4,8*,*15–17*). BAP708 spores were at least 95% pure as judged by light microscopy measuring at least 100 particles per spore preparation. BAP708 spores were unclumped individual spores as judged after evaluating at least 100 spores with light microscopy and at least 500 spores with particle analysis via the Beckman Coulter Multisizer.

References

1. Stewart GS, Johnstone K, Hagelberg E, Ellar DJ. Commitment of bacterial spores to germinate. A measure of the trigger reaction. Biochem J. 1981;198:101–6. PubMed http://dx.doi.org/10.1042/bj1980101

2. Atrih A, Foster SJ. Analysis of the role of bacterial endospore cortex structure in resistance properties and demonstration of its conservation amongst species. J Appl Microbiol. 2001;91:364–72. PubMed http://dx.doi.org/10.1046/j.1365-2672.2001.01394.x

3. Buhr TL, McPherson DC, Gutting BW. Analysis of broth-cultured *Bacillus atrophaeus* and *Bacillus cereus* spores. J Appl Microbiol. 2008;105:1604–13. PubMed http://dx.doi.org/10.1111/j.1365-2672.2008.03899.x

4. Buhr TL, Young AA, Minter ZA, Wells CM, McPherson DC, Hooban CL, et al. Test method development to evaluate hot, humid air decontamination of materials contaminated with *Bacillus anthracis* ΔSterne and B. thuringiensis Al Hakam spores. J Appl Microbiol. 2012;113:1037–51. PubMed http://dx.doi.org/10.1111/j.1365-2672.2012.05423.x

5. Gladstone GP, Fildes P. A simple culture medium for general use without meat extract or peptone. Br J Exp Pathol. 1940;21:161–73.

6. McCartt AD, Gates SD, Jeffries JB, Hanson RK, Joubert LM, Buhr TL. Response of *Bacillus thuringiensis* Al Hakam endospores to gas dynamic heating in a shock tube. Physical Chemistry. 2011;225:1367–77.

7. Buhr TL, Young AA, Minter ZA, Wells CM, Shegogue DA. Decontamination of a hard surface contaminated with *Bacillus anthracis* ΔSterne and *B. anthracis* Ames spores using electrochemically generated liquid-phase chlorine dioxide (eClO2). J Appl Microbiol. 2011;111:1057–64. PubMed http://dx.doi.org/10.1111/j.1365-2672.2011.05122.x

8. Camp DW, Montgomery NK. How good labs can get wrong results—keys to accurate and reproducible quantitation of *Bacillus anthracis* spore sampling or extraction efficiency. Third National Conference on Environmental Sampling and Detection for Biol-Threat Agents; 2008 Dec 2-4; Las Vegas, NV, USA.

9. Doyle RJ, Fariboz N-H, Singh JS. Hydrophobic characteristics of *Bacillus* spores. Curr Microbiol. 1984;10:320–32. http://dx.doi.org/10.1007/BF01626560

10. Koshikawa T, Yamazaki M, Yoshimi M, Ogawa S, Yamada A, Watabe K, et al. Surface hydrophobicity of spores of *Bacillus* spp. J Gen Microbiol. 1989;135:2717–22. PubMed

11. Husmark U, Rönner U. Forces involved in adhesion of *Bacillus cereus* spores to solid surfaces under different environmental conditions. J Appl Bacteriol. 1990;69:557–62. PubMed http://dx.doi.org/10.1111/j.1365-2672.1990.tb01548.x

12. Rönner U, Husmark U, Henriksson A. Adhesion of *bacillus* spores in relation to hydrophobicity. J Appl Bacteriol. 1990;69:550–6. PubMed http://dx.doi.org/10.1111/j.1365-2672.1990.tb01547.x

13. Faille C, Jullien C, Fontaine F, Bellon-Fontaine MN, Slomianny C, Benezech T. Adhesion of *Bacillus* spores and *Escherichia coli* cells to inert surfaces: role of surface hydrophobicity. Can J Microbiol. 2002;48:728–38. PubMed http://dx.doi.org/10.1139/w02-063

14. Charlton S, Moir AJ, Baillie L, Moir A. Characterization of the exosporium of *Bacillus cereus*. J Appl Microbiol. 1999;87:241–5. PubMed http://dx.doi.org/10.1046/j.1365-2672.1999.00878.x

15. Buhr TL, Wells CM, Young AA, Minter ZA, Johnson CA, Payne AN, et al. Decontamination of materials contaminated with *Bacillus anthracis* and *Bacillus thuringiensis* Al Hakam spores using PES-Solid, a solid source of peracetic acid. J Appl Microbiol. 2013;115:398–408. PubMed http://dx.doi.org/10.1111/jam.12253

16. Buhr TL, Young AA, Barnette HK, Minter ZA, Kennihan NL, Johnson CA, et al. Test methods and response surface models for hot, humid air decontamination of materials contaminated with dirty spores of *Bacillus anthracis* ΔSterne and *Bacillus thuringiensis* Al Hakam. J Appl Microbiol. 2015;119:1263–77. PubMed http://dx.doi.org/10.1111/jam.12928

17. Buhr TL, Young AA, Bensman M, Minter ZA, Kennihan NL, Johnson CA, et al. Hot, humid air decontamination of a C-130 aircraft contaminated with spores of two acrystalliferous *Bacillus thuringiensis* strains, surrogates for *Bacillus anthracis*. J Appl Microbiol. 2016;120:1074–84. PubMed http://dx.doi.org/10.1111/jam.13055

Technical Appendix Table. Delineation of the deletion endpoints in BAP417 (BA500 derivative)

Deletion allele	Plasmid	Upstream homology, bp	Downstream homology, bp	Total size of gene, bp	Size of deletion	Remaining sequence
Δcya	pRP1110	447	450	2403	2397	ATG-CAATTG-TAA
Δlef	pRP1091	508	492	2430	2424	ATG-CAATTG-TAA
ΔpagA	pRP1101	1078	1011	2295	2247	ATG-11 codons-GAATTC-3 codons-TAA

			Pathogenicity		
**Ba Strain Name**	**Genetic make up**	**Status/Risk**	**Toxin Genes**	**Capsule Genes**	**All Assay Targets?**
Bacillus anthracis Ames	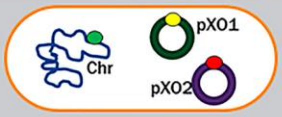	Select Agent	X	X	Yes (3)
Bacillus anthracis Sterne	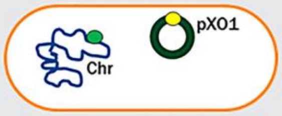	Exempt (pathogenic for animals)	X	-	No (2)
Bacillus anthracis Sterne ΔpXO1 (aka TKO)	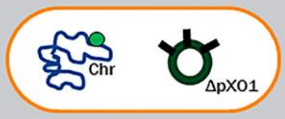	Exempt (non-pathogenic)	-	-	No (1)
Bacillus anthracis Sterne ΔpXO1 plus (rBaSwAT)	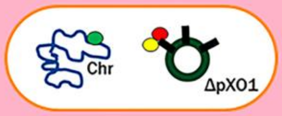	Exempt (non-pathogenic)	-	-	**Yes (3)**

Assay target sequences: (yellow) (red) (green) Gene deletion: ▮ **TKO- Triple Knock Out**

Technical Appendix Figure 1. Schematic representation of wild-type, various mutants, and the recombinant surrogate strain constructed in this study. The genetic properties and pathogenicity of the strains are highlighted in the depiction.

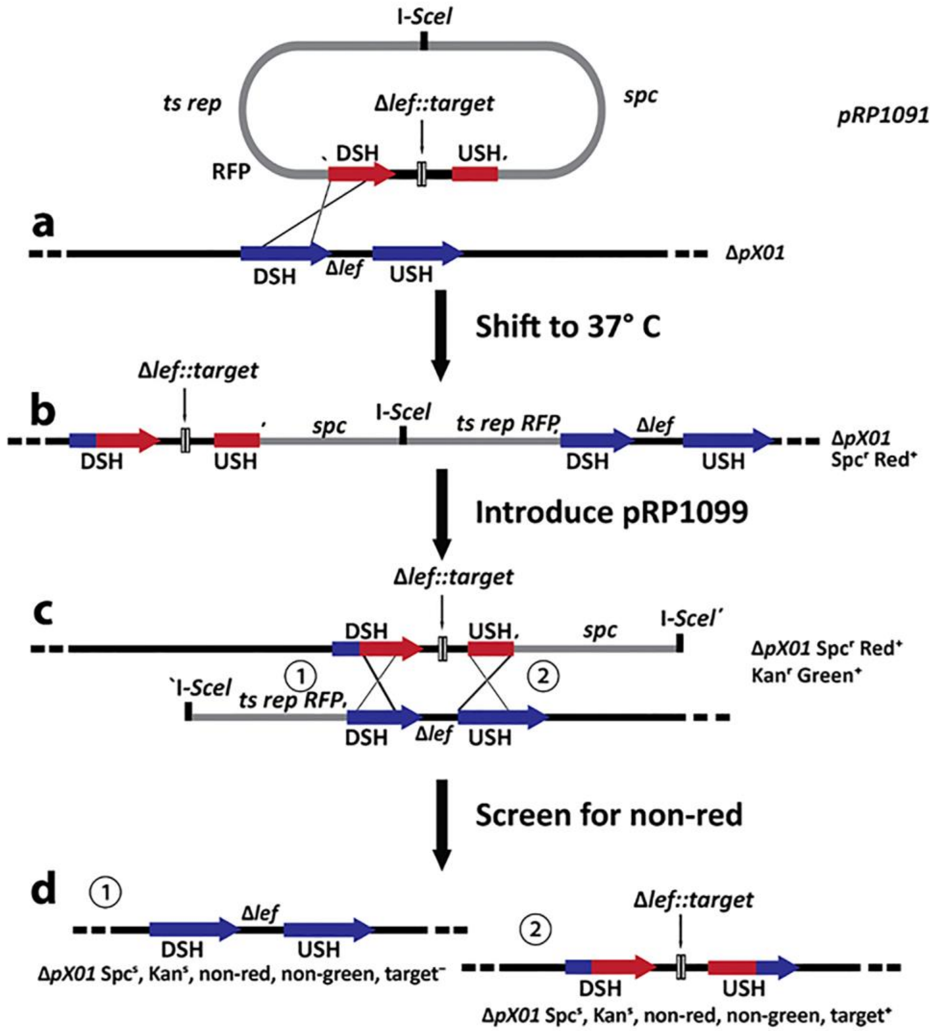

Technical Appendix Figure 2. Schematic of allelic exchange. The various steps depicted are as follows: A) A derivative of pRP1091 containing the insert between the upstream and downstream sequences of *lef* deletion is introduced into BAP417 (triple toxin deletion strain) by biparental mating. The precise boundaries of toxin gene deletions in BAP417 and the upstream and downstream homologies in pRP1091 are listed in the Technical Appendix Table, and the exact procedure for conjugation and transfer of inserts is described in detail elsewhere (*8*). B) Following temperature shift, which prevents plasmid replication, integration of the recombinant pRP1091 derivative plasmid into ΔpXO1 is achieved by homologous recombination by 1 end of the homology (single crossover). C) Introduction of a plasmid (pRP1099) that encodes the enzyme I-*SceI*, which upon expression creates double strand breaks in the co-integrated plasmid and stimulates the second crossover event. D) Resolution of the co-integrate to produce the desired recombinant products. Passage of strains in the absence of kanamycin leads to loss of pRP1099, and screening for nonfluorescent colonies leads to either the restoration of wild-type

sequences (1) or isolation of the recombinant carrying the synthetic cassette (2). The selection and screening of ex-conjugants and resolved products using a combination of antimicrobial drugs and fluorescence markers (TurboRFP and AmCyan) respectively makes this procedure efficient, facile, and user-friendly. Successful insertion of the cassette into ∆pXO1 at the desired location was confirmed by PCR, Sanger sequencing, and whole-genome sequencing. Target, PCR target sequences; DSH, downstream homology; USH, upstream homology.
